# Association between smoking and in-hospital mortality in patients with left ventricular dysfunction undergoing coronary artery bypass surgery: a propensity-matched study

**DOI:** 10.1186/s12872-021-02056-9

**Published:** 2021-05-12

**Authors:** Hanwei Tang, Jianfeng Hou, Kai Chen, Xiaohong Huang, Sheng Liu, Shengshou Hu

**Affiliations:** 1 Department of Cardiovascular Surgery, National Center for Cardiovascular Diseases, State Key Laboratory of Cardiovascular Disease, Fuwai Hospital, Chinese Academy of Medical Sciences and Peking Union Medical College, 167A Beilishi Rd, Xi Cheng District, Beijing, 100037 People’s Republic of China; 2 Department of Special Medical Treatment Center, Fuwai Hospital, National Center for Cardiovascular Diseases, Chinese Academy of Medical Science and Peking Union Medical College, Beijing, People’s Republic of China

**Keywords:** Smoking paradox, CABG, Left ventricular dysfunction, Propensity matched

## Abstract

**Background:**

Data on the effect of smoking on In-hospital outcome in patients with left ventricular dysfunction undergoing coronary artery bypass graft (CABG) surgery are limited. We sought to determine the influence of smoking on CABG patients with left ventricular dysfunction.

**Methods:**

A retrospective study was conducted using data from the China Heart Failure Surgery Registry database. Eligible patients with left ventricular ejection fraction less than 50% underwent isolated CABGS were included. In addition to the use of multivariate regression models, a 1–1 propensity scores matched analysis was performed. Our study (n = 6531) consisted of 3635 smokers and 2896 non-smokers. Smokers were further divided into ex-smokers (n = 2373) and current smokers (n = 1262).

**Results:**

The overall in-hospital morality was 3.9%. Interestingly, current smokers have lower in-hospital mortality than non-smokers [2.3% vs 4.9%; adjusted odds ratio (OR) 0.612 (95% CI 0.395–0.947) ]. No difference was detected in mortality between ex-smokers and non-smokers [3.6% vs 4.9%; adjusted OR 0.974 (0.715–1.327)]. No significant differences in other clinical end points were observed. Results of propensity-matched analyses were broadly consistent.

**Conclusions:**

It is paradoxically that current smokers had lower in-hospital mortality than non-smokers. Future studies should be performed to further understand the biological mechanisms that may explain this ‘smoker’s paradox’ phenomenon.

**Supplementary Information:**

The online version contains supplementary material available at 10.1186/s12872-021-02056-9.

## Background

Smoking has been well documented as a major risk factor for coronary heart disease (CAD) and premature death [1, 2]. Smoking is also associated with development of left ventricular dysfunction [3]. However, in 1973, Helmers et al. reported that smokers had a lower risk of mortality than non-smokers [4]. Some subsequent studies also showed this so called ‘smoker’s paradox’ phenomenon in different aspects [3, 5–7]. Coronary artery bypass grafting surgery (CABG) is an important method of treatment of CAD. From a surgical point of view, the impact of smoking on long-term postoperative outcomes have been reported [2, 8], while the association between smoking and in-hospital mortality in CABG patients remains controversial. Few limited studies have failed to show the adverse impact of smoking on in-hospital mortality [9, 10]. The main risk scoring systems in cardiac surgery such as EuroScore and the Society of Thoracic Surgeons risk models also failed to demonstrate the effect of active smoking on operative mortality [11]. Therefore, controversies still exist on this topic. Moreover, data on the effect of smoking on short-term outcome in patients with left ventricular dysfunction undergoing CABGs remain scarce.

It is of important to examine the true effect of smoking on outcome among contemporary patients with left ventricular dysfunction undergoing CABG. The phenomenon of ‘smoking paradox’ has a negative effect on smoking cessation in a public health perspective and surgeon’s practice. In the current study, we aim to examine the effect of different smoking status on the postoperative outcomes of patients with left ventricular dysfunction receiving isolated CABGs.

## Methods

### Study design

**Fig. 1:**
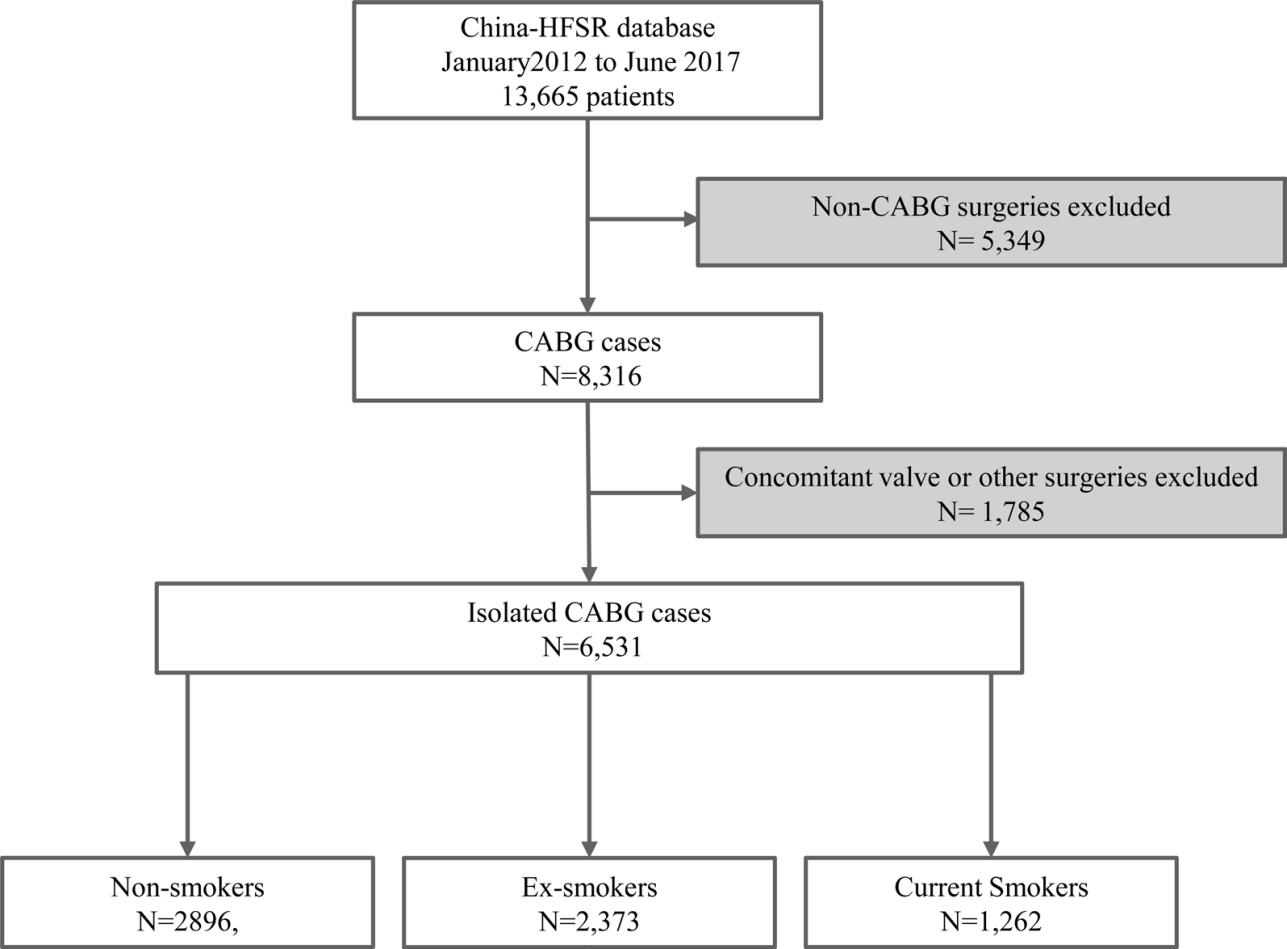
Patients flow chart. From January 2012 to June 2017, 13,665 patients aged > 17 years were registered in China-HFSR databases. Those received Non-CABG surgeries (n = 5349) or CABGs concomitant with valve or other surgeries (n = 1785) were excluded. The final cohort included 6531 patients

The China Heart Failure Surgery Registry (China-HFSR) was led by Fuwai Hospital and other representative cardiac centres in different regions around China. All centres participate in China-HFSR had annual cardiac surgery volume > 100 cases. In total, 94 centres covering a broad geographic region were included as participants in the study. We included patients ≥ 17 years old who underwent CABG from January 2012 to June 2017 with documented left ventricular ejection fraction (LVEF) < 50%. Patients were excluded if they underwent concomitant valve or other surgeries. These patients were then stratified according to preoperative smoking status (Fig. 1). Current smokers were defined as those who smoked within 1 month before admission. Ex-smokers were defined as those who quitted smoking for at least 1 month. Non-smokers were defined as those who never smoked. All CABG procedures represented standard surgical approaches to surgical myocardial revascularization with and without the use of cardiopulmonary bypass support. This study was approved by the institutional review board at Fuwai Hospital (approval number 887, April 25th, 2017) and carried out in accordance with relevant guidelines and regulations. The informed consent was signed by participants.

### Data collection

All data were collected at the local sites from the medical records. The requirements for data collection and the definitions of variables were clearly identified. All data were entered into the database separately by two trained technicians using standardized electronic case report forms at the local sites and then submitted online to the data processing centre. Two separate reviewers from the data processing centre randomly selected and assessed 5–10% of each of the participating centres’ medical records during annual on-site audits. We compared the data in the database and the original medical records. A committee composed of physicians and surgeons determined the correct final value when there was a disagreement. In all patients included in China-HFSR database, 91 (1.4%) of them s don’t have height data, and 78 (1.2%) of which were without weight. Considering the fact that they only accounted for a very small proportion of our patients, we imputed missing continuous variables (height and weight) with different mean values for different sex gender.

### Clinical data

The preoperative variables including age, gender, body mass index, New York Heart Association (NYNH) classification, Canadian Cardiovascular Society (CCS) classification, diabetes mellitus (DM), hypertension, hyperlipidaemia, renal failure, chronic obstructive pulmonary disease (COPD), cerebrovascular accident, carotid disease and other peripheral arterial disease, preoperative atrial fibrillation, previous myocardial infarction (MI), percutaneous transluminal coronary angioplasty (PTCA) history, Number of diseased vessels, left main CAD, LVEF, preoperative creatinine and prior cardiovascular surgeries. Data regarding preoperative intra-aortic balloon pump (IABP) insertion, operative priority and cardiopulmonary bypass using were also collected.

The major postoperative complications included re-intubation, prolonged ventilation (> 24 h), MI, mediastinal infection, stroke, renal failure, multiple organ dysfunction syndrome and reoperation for bleeding. MI was counted as a complication if it newly occurred postoperatively meet the following criteria (≥ 1): (1) MI documented in the medical record with an elevation of cardiac troponin values with at least one value above the 10 times 99th percentile upper reference limit; (2) electrocardiograph-documented ST-segment elevation in evolution, Q waves 0.03 s in width and/or one-third or greater of the total QRS complex in 2 or more contiguous leads; (3) new left bundle branch block [12]. Mediastinal infection was defined according to the published expert consensus [13]. Stroke was defined as a central neurological deficit persisting > 24 h (i.e., extremity weakness or loss of motion, loss of consciousness, loss of speech, visual field cuts). Renal failure was defined as an increase in serum creatinine level to > 4 mg/dL, 3 times the most recent preoperative creatinine level, or a new postoperative need for dialysis. Reoperation for bleeding was defined as chest tube drainage ≥ 200 mL/h for at least 3 h requiring surgical intervention.

### Statistical analysis

Continuous variables are expressed as either mean ± standard deviation (SD) or medians and interquartile range (IQR) depending upon variable distribution. Categorical variables are presented as frequencies and percentages. We performed a t-test for normally distributed continuous variables; otherwise, the Mann–Whitney U test or Kruskal–Wallis H test was used. This was followed by the Bonferroni t-test with a corrected *P* value of 0.05/3. Chi square tests or Fisher’s exact tests were used for categorical variables.

We used the following 2 techniques to adjust for potential confounders when comparing outcomes of the different smoking status groups: multiple logistic regression modelling and propensity matching. For the regression-based analyses, the association between smoking status and each clinical end point were adjusted for baseline patient risk by inclusion of the following validated and widely accepted measures of patient-level covariates: age, body mass index, sex, diabetes mellitus, hypertension, hyperlipidemia, chronic renal failure, chronic obstructive pulmonary disease, cerebrovascular accident, carotid disease, peripheral artery disease, previous MI, PTCA history, LVEF, preoperative creatinine, CCS classification, NYHA classification, triple vessel disease, Left main CAD, preoperative IABP, operative priority, off-pump technique and prior cardiovascular history. Model results are reported as odds ratios (OR) with a 95% confidence interval (CIs).

The second method of adjusting for potential confounders involved matching patients with similar estimated probability of smoking status (propensity score). The propensity score was calculated by a multivariable logistic regression model which was developed using the same covariates listed above for the regression-based analyses. Then we matched patients in a 1:1 fashion without replacement [14]. We performed PS matching between ex-smokers and non-smokers, and between current and non-smokers. ORs with 95% CIs comparing the frequency of each end point for ex-smokers vs non-smokers and current smokers versus non-smokers were estimated using univariable logistic regression.

Additional analysis were performed to examine whether the association between smoking status and mortality differed across subgroups based on age, sex, ejection fraction, diabetes mellitus, hypertension and chronic lung disease. Subgroup-specific ORs were estimated and displayed with 95% CIs.

All reported *P* values are 2 sided, and values of *P* < 0.05 were considered to indicate statistical significance.When applying for multiple comparison, a Bonferroni adjustment with a corrected *P* value of 0.0167 (0.05/3) was introduced. All statistical analysis was performed using SPSS version 22.0 (IBM Corp., Armonk, NY).

## Results

### Baseline characteristics

Of all patients, 55.7% (3635) patients had smoking history and 96.5% (6303) patients received elective surgeries. Smokers were further divided into ex-smokers (n = 2373) and current smokers (n = 1262). Baseline characteristics before matching are presented in Table 1.

**Table 1:** Baseline demographic and clinical characteristics in overall population

Variable	All patients (n = 6531)	Smoking status			*P*
		Non-smokers (n = 2896)	Ex-smokers (n = 2373)	Current smokers (n = 1262)	
Age, mean (SD), years	61.4 (9.2)	62.8 (9.3)	60.7 (8.9)	59.6 (9.1)	< 0.001
Female, n (%)	1060 (16.2)	882 (30.5)	117 (4.9)	61 (4.8)	< 0.001
BMI, median (quartile)	24.5 (22.6, 26.7)	24.4 (22.5, 26.5)	24.6 (22.8, 27.0)	24.7 (22.8, 26.9)	0.001
Diabetes mellitus, n (%)	2225 (34.1)	1067 (36.8)	674 (28.4)	484 (38.4)	< 0.001
Hypertension, n (%)	3571 (54.7)	1629 (56.3)	1245 (52.5)	697 (55.2)	0.021
Hyperlipemia, n (%)	2101 (32.2)	684 (23.6)	834 (35.1)	583 (46.2)	< 0.001
Chronic renal failure, n (%)	128 (2.0)	73 (2.5)	37 (1.6)	18 (1.4)	0.014
COPD, n (%)	108 (1.7)	39 (1.3)	51 (2.1)	18 (1.4)	0.059
Peripheral artery disease, n (%)	281 (4.3)	119 (4.1)	129 (5.4)	33 (2.6)	< 0.001
Carotid disease, n (%)	1061 (16.2)	443 (15.3)	428 (18.0)	190 (15.1)	0.012
Cerebrovascular accident, n (%)	576 (8.8)	290 (10.0)	177 (7.5)	109 (8.6)	0.005
Creatinine, median (quartile), umol/dL	82.0 (70.0, 97.0)	80.6 (67.0, 96.0)	83.0 (72.0, 98.0)	84.0 (72.2, 96.9)	0.583
Left main CAD, n (%)	1800 (27.6)	907 (31.3)	569 (24.0)	324 (25.7)	< 0.001
Triple vessel disease, n (%)	4531 (69.4)	2284 (78.9)	1499 (63.2)	748 (59.3)	< 0.001
Previous MI, n (%)	2787 (42.7)	1110 (38.3)	1112 (46.9)	565 (44.6)	< 0.001
PTCA history, n (%)	844 (12.9)	402 (13.9)	295 (12.4)	147 (11.6)	0.096
*CCS class*					< 0.001
NA, n (%)	1274 (19.5)	584 (20.2)	462 (19.5)	228 (18.1)	
I, n (%)	914 (14.0)	514 (17.7)	267 (11.3)	133 (10.5)	
II, n (%)	2196 (33.6)	945 (32.6)	851 (35.9)	400 (31.7)	
III, n (%)	1720 (26.3)	718 (24.8)	621 (26.2)	381 (30.2)	
IV, n (%)	427 (6.5)	135 (4.7)	172 (7.2)	120 (9.5)	
LVEF, Mean (SD), %	42.1 (5.3)	42.3 (5.4)	42.1 (5.3)	41.9 (5.4)	0.09
*NYHA class*					< 0.001
I, n (%)	938 (14.4)	387 (13.4)	387 (16.3)	164 (13.0)	
II, n (%)	2365 (36.2)	1016 (35.1)	893 (37.6)	456 (36.1)	
III, n (%)	2846 (43.6)	1340 (46.3)	943 (39.7)	563 (44.6)	
IV, n (%)	382 (5.8)	153 (5.3)	150 (6.3)	79 (6.3)	
Prior cardiovascular surgery, n (%)	86 (1.3)	34 (1.2)	36 (1.5)	16 (1.3)	0.546
Elective surgery, n (%)	6303 (96.5)	2811 (97.1)	2282 (96.2)	1210 (95.9)	0.083
Preoperative IABP, n (%)	196 (3.0)	88 (3.0)	62 (2.6)	46 (3.6)	0.219
Number of graft, Median (quartile)	4 (3, 4)	4 (4, 5)	4 (3, 4)	4 (3, 4)	< 0.001
Off-pump surgery, n (%)	1224 (18.7)	328 (11.3)	583 (24.6)	313 (24.8)	< 0.001
*EuroSCORE*					< 0.001
0–2, n (%)	1626 (24.9)	500 (17.3)	714 (30.1)	412 (32.6)	
3–5, n (%)	3323 (50.9)	1525 (52.7)	1200 (50.6)	598 (47.4)	
6 plus, n (%)	1582 (24.2)	871 (30.1)	459 (19.3)	252 (20.0)	

*BMI* body mass index, *CAD* coronary vascular disease, *CCS* Canadian Cardiovascular Society, *COPD* chronic obstructive pulmonary disease, *IABP* intra-aortic balloon pump, *LFEF* left ventricular ejection fraction, *MI* myocardial infarction, *NA* not available, *NYHA* New York Heart Association, *PTCA* percutaneous transluminal coronary angioplasty, *SD* standard deviation

Current smokers were slightly younger (59.6 ± 9.1 vs 62.8 ± 9.3 years) with higher proportion of male patients (95.2% vs 69.5%) compared with non-smokers. The incidence of hyperlipidaemia (46.2% vs 23.6%), previous MI (44.8% vs 38.3) and CCS class III/IV (39.7% vs29.5%) were higher in current smokers compared with non-smokers. Compared with non-smokers, current smokers were less likely to have left main CAD (25.7% vs 31.3%) and triple vessel disease (59.3% vs 78.9%). Among ex-smokers, the proportion of peripheral artery disease was higher than non-smokers (5.4% vs 2.6%). The non-smokers were less likely to receive off-pump CABG than ex-smokers (11.3% vs 24.6%) and current smokers (11.3% vs 24.8%).

### Operative outcomes

Table 2 summarizes the outcomes from the unmatched groups. The overall in-hospital morality was 3.9%. Interestingly, current smokers have lower in-hospital mortality than non-smokers [2.3% vs 4.9%; adjusted OR 0.612 (95% CI 0.395–0.947)]. No difference was detected in mortality between ex-smokers and non-smokers [3.6% vs 4.9%; adjusted OR 0.974 (0.715–1.327)]. No significant differences in other clinical end points were observed.

**Table 2:** Number of end point events and covariate-adjusted ORs in overall population

End Point	No. (%) of events by group			OR (95% CI)^a^	
	Non-smokers (n = 2896)	Ex-smokers (n = 2373)	Current smokers (n = 1262)	Ex-smokers (n = 2373)	Current smokers (n = 1262)
Mortality	142 (4.9)	86 (3.6)	29 (2.3)	0.974 (0.715–1.327)	0.612 (0.395–0.947)
Re-intubation	88 (3.0)	56 (2.4)	28 (2.2)	0.887 (0.613–1.282)	0.911 (0.575–1.442)
Postoperative MI	33 (1.1)	21 (0.9)	11 (0.9)	0.778 (0.425–1.421)	0.712 (0.341–1.487)
Mediastinal infection	34 (1.2)	13 (0.5)	4 (0.6)	0.599 (0.299–1.199)	0.570 (0.240–1.355)
Postoperative stroke	19 (0.7)	10 (0.4)	5 (0.4)	0.810 (0.351–1.874)	0.756 (0.265–2.153)
Postoperative renal failure	73 (2.5)	51 (2.1)	19 (1.5)	0.941 (0.629–1.409)	0.650 (0.377–1.121)
Ventilation > 24 h	279 (9.6)	235 (9.9)	110 (8.7)	1.081 (0.877–1.332)	0.819 (0.749–1.256)
MODS	61 (2.1)	27 (1.1)	12 (1.0)	0.704 (0.426–1.164)	0.629 (0.324–1.221)
Re-operation	66 (2.3)	58 (2.4)	24 (1.9)	1.070 (0.721–1.587)	0.891 (0.539–1.474)

*CI* confidence interval, *MI* myocardial infarction, *MODS* multiple organ dysfunction syndrome, *OR* odds ratio

^a^Non-smokers as reference

**Fig. 2:**
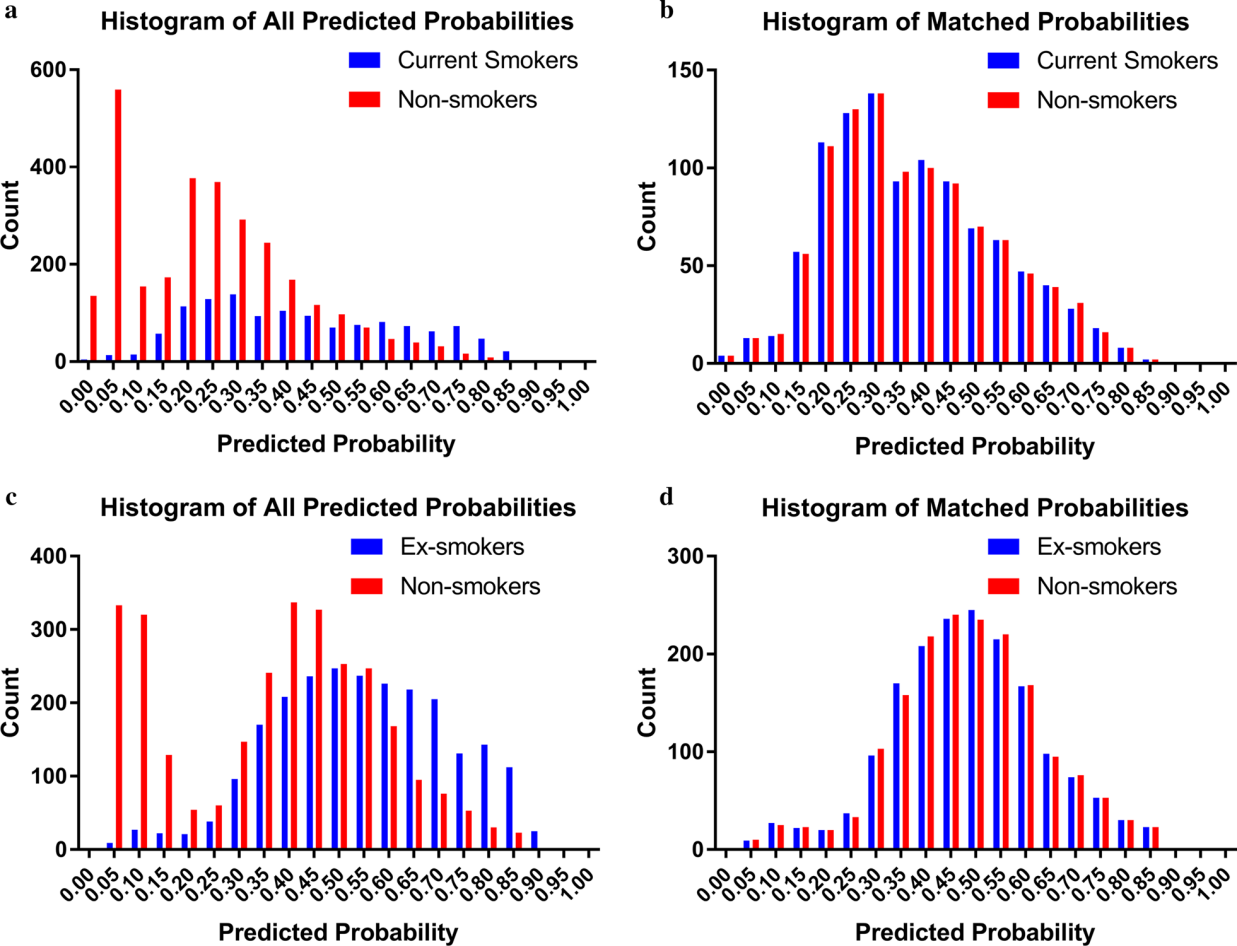
**a** Preoperative characteristics varied widely between non-smokers and current smokers. **b** After matching, there were no significant differences between the matched cohorts (non-smokers vs current smokers), **c** Preoperative characteristics varied widely between non-smokers and ex-smokers. **d** After matching, there were no significant differences between the matched cohorts (non-smokers vs ex-smokers)

After propensity matching, 2 comparable groups [1731 of each for non-smokers vs ex-smokers (Additional file 1: Table S1) and 1032 of each for non-smokers vs current smokers (Additional file 1: Table S2)] were created (Fig. 2). Outcomes for propensity-matched patients are displayed in Table 3 (non-smokers vs ex-smokers) and Table 4 (non-smokers vs current smokers). Operative mortality was similar for non-smokers vs ex-smokers [5.2% vs 4.3%; OR 0.814 (95% CI 0.594–1.116)]. Less mortality was found in current smokers (2.8%) compared with non-smokers [4.9%; OR 0.556 (95% CI 0.350–0.885)]. For the other clinical end points, no significant differences were found.

**Table 3:** Number of end point events and ORs in propensity-matched group (non-smokers vs ex-smokers)

End Point	No. (%) of events by group		OR (95% CI)	*P*
	Non-smokers (n = 1731)	Ex-smokers (n = 1731)		
Mortality	90 (5.2)	74 (4.3)	0.814 (0.594–1.116)	0.201
Re-intubation	65 (3.8)	51 (2.9)	0.778 (0.536–1.130)	0.187
Postoperative MI	22 (1.3)	19 (1.1)	0.862 (0.465–1.599)	0.638
Mediastinal infection	16 (0.9)	11 (0.6)	0.686 (0.317–1.481)	0.337
Postoperative stroke	10 (0.6)	7 (0.4)	0.699 (0.265–1.840)	0.468
Postoperative renal failure	44 (2.5)	45 (2.6)	1.023 (0.672–1.559)	0.914
Ventilation > 24 h	171 (9.9)	177 (10.2)	1.039 (0.388–1.297)	0.735
MODS	34 (2.0)	24 (1.4)	0.702 (0.414–1.188)	0.188
Re-operation	46 (2.7)	48 (2.8)	1.045 (0.993–1.574)	0.834

*CI* confidence interval, *MI* myocardial infarction, *MODS* multiple organ dysfunction syndrome, *OR* odds ratio

**Table 4:** Number of end point events and ORs in propensity-matched group (non-smokers vs current smokers)

End Point	No. (%) of events by group		OR (95% CI)	*P*
	Non-smokers (n = 1032)	Current smokers (n = 1032)		
Mortality	51 (4.9)	29 (2.8)	0.556 (0.350–0.885)	0.013
Re-intubation	43 (4.2)	27 (2.6)	0.618 (0.379–1.008)	0.054
Postoperative MI	14 (1.4)	10 (1.0)	0.711 (0.315–1.609)	0.414
Mediastinal infection	13 (1.3)	7 (0.7)	0.535 (0.213–1.347)	0.184
Postoperative stroke	6 (0.6)	5 (0.5)	0.833 (0.253–2.736)	0.763
Postoperative renal failure	19 (1.8)	17 (1.6)	0.893 (0.461–1.728)	0.737
Ventilation > 24 h	97 (9.4)	95 (9.2)	0.977 (0.726–1.315)	0.880
MODS	17 (1.6)	12 (1.2)	0.702 (0.334–1.478)	0.702
Re-operation	23 (2.2)	23 (2.2)	1.000 (0.557–1.794)	1.000

*CI* confidence interval, *MI* myocardial infarction, *MODS* multiple organ dysfunction syndrome, *OR* odds ratio

### Subgroup analysis

Effect of smoking status among patient subgroups in the propensity matched cohorts was examined. For each subgroup in non-smokers vs ex-smokers, all the calculations include 1.0 in the 95% CI for the OR and the interaction *P* value was not significant (*P* ≥ 0.05) (Additional file 1: Table S3). Of non-smokers vs current smokers, current smokers were found to have lower mortality in patients younger than 65 years [4.6% vs 2.4%; OR 0.503 (95% CI 0.277–0.915)] and patients without COPD (2.9% vs 4.9%; OR 0.566 (95% CI 0.355–0.902)] (Additional file 1: Table S4).

## Discussion

The present study reports upon the effect of preoperative smoking status on the operative outcomes of CABG. We performed analyses on the unmatched and propensity-matched cohorts, controlling for the preoperative risk factors. In our study of CABG patients with left ventricular dysfunction, we have not seen a protective effect of smoking in terms of postoperative complications. However, it is paradoxically that current smokers had lower in-hospital mortality than non-smokers, particularly in patients younger than 65 years or patients without COPD.

Smoking substantially increases the risk of developing CAD and HF in men and women [15, 16]. It is estimated that 16.5% of all deaths in men and 1.7% in women were due to smoking in China [17]. In an analysis of the studies of left ventricular dysfunction (SOLVD) database, current smoking was found to be a risk factor of mortality and morbidity in patients with left ventricular dysfunction [18]. The constituents of inhaled tobacco contributing to multiple adverse effect on cardiovascular system like endothelial dysfunction, platelet dysfunction, increased heart rate, increased coagulation, increased blood pressure, increased myocardial oxygen demand and vasoconstriction [3, 19, 20]. These adverse effects might be much more pronounced in patients with impaired ventricular function. Of cardiac surgical patients, some prior studies have reported the deleterious effect of smoking on pulmonary complications [10, 11]. Unfortunately, studies in cardiac surgical patients with impaired left ventricular function remain scarce.

In our analysis, current smoking does not seem to have bearing on postoperative complications and it is paradoxically that current smokers had lower in-hospital mortality than non-smokers. This in part might be due to the difference in preoperative characteristics. Compared with non-smokers, smokers may develop CAD and experience CAD symptoms much earlier in the course of their disease, at a time when their prognosis tends to be still more favourable. In this study, current smokers were slightly younger and did have less left main CAD and triple vessel disease. Difference in clinical outcomes potentially reflects intrinsic differences in risk and in-hospital care.

Fonarow and colleagues put forward a hypothesis that abrupt cessation of smoking during the HF hospitalization improves outcomes among smokers [3]. This theory may also holds true in CABG patients with left ventricular dysfunction. In China, most patients will be admitted to hospitals a couple of days prior to operation (above 1 week) for preoperative evaluation and preparation. It is then that the abrupt cessation of smoking during the hospitalization allows for the rapid stabilization of the balance between oxygen demand and supply. Suskin et al. reported that quitting smoking was associated with a decrease in morbidity and mortality in patients with left ventricular dysfunction [18]. Moreover, the abrupt smoking cessation may also contribute in recovery of airway function [11]. A prospective study from Warner and colleagues showed the benefit effect of a short period of smoking cessation on operative outcomes [21]. As Benedetto et al. reported, smoking cessation reduced the risk for all pulmonary complications [11].

In the current studies, the incidence of COPD in smokers was much lower than studies in western countries [9, 11, 22]. The low incidence of COPD in Chinese surgical patients may partly explain the results of current study. In 2003, Arabaci et al. reported smoking caused obstructive type respiratory problems and worsening of existing restrictive respiratory problems [23]. As many current smokers may not suffer from the worsening of existing respiratory problems during the perioperative period, adverse outcomes may not present during the in-hospital stay.

In subgroup analysis, we detected current smokers have lower mortality compared with non-smokers in patients younger than 65 years rather than in elderly patients. He and colleagues reported smoking history as an independent predictor of mortality in the elderly patients [24]. Published in 2011, Jones and colleagues found current smokers had a higher risk of adverse outcomes of cardiac surgery in the elderlys [25]. Hence it is of important to encourage elderly patients to quit smoking before surgery as these patients may be vulnerable to postoperative complications.

The apparent smoker’s paradox should not be interpreted as a benefit of or justification for smoking. We still advocate that patients stop smoking. In a 20-year follow-up study from van Domburg and colleagues, smoking cessation reduces long-term mortality and repeat revascularization procedures after CABG [8]. A recent study from Masoudkabir et al. showed smoking cessation after CABG could reduce the 5-year mortality by 35% [2]. Shahim and colleagues found current smokers had a higher risk of 5-year adverse outcomes [26]. Smoking is established as an independent risk factor for mortality and recurrent MI [27]. From a prevention perspective, cigarette smoking is a well-established risk factor for cardiovascular disease and death [17, 28] which smoking cessation and controlling programs should be promoted in the whole society.

In spite of the public gradually raised awareness of the hazards of smoking and Chinese government’s effort in the tobacco control, there is still a high prevalence of smoking in current China [17]. One-third of the world’s tobacco is produced and consumed in China [29]. The smoker’s paradox whereby smokers have an increased risk of cardiovascular diseases but have lower mortality for in-hospital HF and PTCA patients has been previously reported [3, 7]. This phenomenon may mislead the public on smoking cessation and many surgeons may ignore the importance of smoking cessation counselling. Understanding the phenomenon of ‘smoking paradox’ is of important in a public health perspective and surgeon’s practice. Future studies are requested to investigate the mechanisms of this phenomenon.

## Limitations

There are few limitations in our work. Firstly, the smoking status was determined on the basis of self-report. We did not use biochemical testing to confirm the accuracy of self-reports. Secondly, detailed smoking status regarding packs per day or duration of smoking was not available in this study. Also, there may be residual measured and unmeasured confounding variables which influence the findings. Lastly, we observed in-hospital mortality rather than standard 30-day mortality. However, within these limitations, we believe our work will provide essential data for assisting clinical practices.

## Conclusions

As a national-wide multicentre study, we provide insights upon the effect of preoperative smoking status on the operative outcomes of CABG in patients with left ventricular dysfunction. Our findings did not show a protective effect of smoking in terms of postoperative complications. However, it is paradoxically that current smokers had lower in-hospital mortality than non-smokers. Future studies should be performed to further understand the biological mechanisms that may explain this phenomenon.

## Supplementary Information

**Additional file 1**. Supplemental Appendices.

## Data Availability

The datasets generated during and analyzed during the current study are not publicly available due to the datasets also forms part of other ongoing studies but are available from the corresponding author on reasonable request.

## Abbreviations

CABG: Coronary artery bypass grafting
CAD: Coronary artery disease
CCS: Canadian Cardiovascular Society
CI: Confidence interval
COPD: Chronic obstructive pulmonary disease
DM: Diabetes mellitus
IABP: Intra-aortic balloon pump
LVEF: Left ventricular ejection fraction
MI: Myocardial infarction
NYHA: New York Heart Association
OR: Odds ratio
PTCA: Percutaneous transluminal coronary angioplasty
