# Supplementary material for: Association between smoking and in-hospital mortality in patients with left ventricular dysfunction undergoing coronary artery bypass surgery: a propensity-matched study
Source: BMC Cardiovasc Disord. 2021 May 12;21:236. doi: 10.1186/s12872-021-02056-9 (PMC8114501; doi:10.1186/s12872-021-02056-9)
Supplement: Supplementary file 1 — Additional file 1. Supplemental Appendices. [file 12872_2021_2056_MOESM1_ESM.docx]

**Supplemental Material**

Association between Smoking and In-hospital mortality in Patients with Left Ventricular Dysfunction Undergoing Coronary Artery Bypass Surgery: A Propensity-matched Study

**Authors:**

Hanwei Tang, MD; Jianfeng Hou, MD, PhD; Kai Chen, MD, PhD; Xiaohong Huang, MD, PhD; Sheng Liu, MD, PhD; Dr. Shengshou Hu, MD, PhD

**Contents**

**Supplemental Tables**

**Table S1.** Baseline Demographic and Clinical Characteristics in Propensity-Matched Group (Non-smokers vs Ex-smokers)

**Table S2.** Baseline Demographic and Clinical Characteristics in Propensity-Matched Group (Non-smokers vs Current smokers)

**Table S3.** Mortality Ors for Subgroups in Propensity-Matched Group (Non-smokers vs Ex-smokers)

**Table S4.** Mortality Ors for Subgroups in Propensity-Matched Group (Non-smokers vs Current smokers)

Table S1 Baseline Demographic and Clinical Characteristics in Propensity-Matched Group (Non-smokers vs Ex-smokers).

|  |  | Smoking Status | |  |
| --- | --- | --- | --- | --- |
| Variable | All Patients  (n = 3462) | Non-smokers  (n=1731) | Ex-smokers  (n=1731) | *P* |
| Age, mean (SD), y | 61.6(9.3) | 61.8(9.7) | 61.4(8.9) | 0.007 |
| Female, n (%) | 214(6.2) | 97(5.6) | 117(6.8) | 0.158 |
| BMI, median (quartile) | 24.5(22.7,26.7) | 24.5(22.8,26.6) | 24.5(22.7,26.8) | 0.574 |
| Diabetes mellitus, n (%) | 1112(32.1) | 554(23.0) | 558(32.2) | 0.884 |
| Hypertension, n (%) | 1850(53.4) | 929(53.7) | 921(53.2) | 0.785 |
| Hyperlipemia, n (%) | 852(24.6) | 411(23.7) | 441(25.5) | 0.237 |
| Chronic renal failure, n (%) | 76(2.2) | 42(2.4) | 34(2.0) | 0.353 |
| COPD, n (%) | 60(1.7) | 31(1.8) | 29(1.7) | 0.795 |
| Peripheral artery disease, n (%) | 172(5.0) | 84(4.9) | 88(5.1) | 0.754 |
| Carotid disease, n (%) | 570(16.5) | 275(15.9) | 295(17.0) | 0.359 |
| Cerebrovascular accident, n (%) | 296(8.5) | 151(8.7) | 145(8.4) | 0.715 |
| Creatinine, median (quartile), umol/dL | 83.0(71.2,98.0) | 84.1(72.0,98.0) | 82.3(71.0,97.7) | 0.080 |
| Left main CAD, n (%) | 998(28.8) | 509(29.4) | 489(28.2) | 0.453 |
| Triple vessel disease, n (%) | 2579(74.5) | 1276(73.7) | 1303(75.3) | 0.292 |
| Previous MI, n (%) | 1431(41.3) | 706(40.8) | 725(41.9) | 0.512 |
| PTCA history, n (%) | 449(13.0) | 220(12.7) | 229(13.2) | 0.649 |
| CCS class |  |  |  | <0.001 |
| NA, n (%) | 712(20.6) | 339(19.6) | 373(21.5) |  |
| I, n (%) | 551(15.9) | 333(19.2) | 218(12.6) |  |
| II, n (%) | 1166(33.7) | 553(31.9) | 613(35.4) |  |
| III, n (%) | 848(24.5) | 428(24.7) | 420(24.3) |  |
| IV, n (%) | 185(5.3) | 78(4.5) | 107(6.2) |  |
| LVEF, Mean (SD), % | 42.0(5.4) | 41.9(5.6) | 42.1(5.3) | 0.892 |
| NYHA class |  |  |  | 0.004 |
| I, n (%) | 479(13.8) | 222(112.8) | 257(14.8) |  |
| II, n (%) | 1264(36.5) | 615(35.2) | 649(37.5) |  |
| III, n (%) | 1515(43.8) | 806(46.6) | 709(14.0) |  |
| IV, n (%) | 204(5.9) | 88(5.1) | 116(6.7) |  |
| Prior cardiovascular surgery, n (%) | 47(1.4) | 22(1.3) | 25(1.4) | 0.660 |
| Elective surgery, n (%) | 3354(96.9) | 1674(96.7) | 1680(97.1) | 0.557 |
| Preoperative IABP, n (%) | 96(2.8) | 45(2.6) | 51(2.9) | 0.535 |
| Number of graft, Median (quartile) | 4 (4, 5) | 4 (4, 4) | 4 (4, 5) | 0.862 |
| Off-pump surgery, n (%) | 465(13.4) | 233(13.5) | 232(13.4) | 0.960 |
| EuroSCORE |  |  |  | 0.599 |
| 0-2, n (%) | 914(26.4) | 450(26.0) | 464(26.8) |  |
| 3-5, n (%) | 1784(51.5) | 887(51.2) | 897(51.8) |  |
| 6 plus, n (%) | 764(22.1) | 394(22.8) | 370(21.4) |  |

BMI indicate body mass index, CAD, coronary vascular disease; CCS, Canadian Cardiovascular Society; COPD, chronic obstructive pulmonary disease; IABP, intra-aortic balloon pump; LFEF, left ventricular ejection fraction; MI, myocardial infarction; NA, not available; NYHA, New York Heart Association; PTCA, percutaneous transluminal coronary angioplasty; SD, standard deviation.

Table S2 Baseline Demographic and Clinical Characteristics in Propensity-Matched Group (Non-smokers vs Current Smokers)

|  |  | Smoking Status | |  |
| --- | --- | --- | --- | --- |
| Variable | All Patients  (n = 2064) | Non-smokers  (n=1032) | Current Smokers  (n=1032) | *P* |
| Age, mean (SD), y | 60.2(9.3) | 60.2(9.7) | 60.3(8.9) | 0.360 |
| Female, n (%) | 101(4.9) | 40(3.9) | 61(5.9) | 0.032 |
| BMI, median (quatile) | 24.6(22.7,26.7) | 24.5(22.8,26.7) | 24.6(22.6,26.8) | 0.740 |
| Diabetes mellitus, n (%) | 746(36.1) | 355(34.4) | 391(37.9) | 0.099 |
| Hypertension, n (%) | 1111(53.8) | 544(52.7) | 567(54.9) | 0.310 |
| Hyperlipemia, n (%) | 695(33.7) | 326(31.6) | 369(35.8) | 0.045 |
| Chronic renal failure, n (%) | 42(2.0) | 25(2.4) | 17(1.6) | 0.212 |
| COPD, n (%) | 35(1.7) | 19(1.8) | 16(1.6) | 0.609 |
| Peripheral artery disease, n (%) | 59(2.9) | 27(2.6) | 32(3.1) | 0.509 |
| Carotid disease, n (%) | 315(15.3) | 154(14.9) | 161(15.6) | 0.668 |
| Cerebrovascular accident, n (%) | 192(9.3) | 93(9.0) | 99(9.6) | 0.649 |
| Creatinine, median (quartile), umol/dL | 84.0(72.0,97.3) | 85.0(72.0,98.4)) | 83.0(72.0,96.0) | 0.104 |
| Left main CAD, n (%) | 569(27.6) | 268(26.0) | 301(29.2) | 0.104 |
| Triple vessel disease, n (%) | 1413(68.5) | 694(67.2) | 719(69.7) | 0.236 |
| Previous MI, n (%) | 992(44.7) | 459(44.5) | 463(44.9) | 0.859 |
| PTCA history, n (%) | 270(13.1) | 129(12.5) | 141(13.7) | 0.433 |
| CCS class |  |  |  | <0.001 |
| NA, n (%) | 404(19.6) | 185(17.9) | 219(21.2) |  |
| I, n (%) | 361(17.5) | 236(22.9) | 125(12.1) |  |
| II, n (%) | 618(29.9) | 308(29.8) | 310(30.0) |  |
| III, n (%) | 550(26.0) | 257(24.9) | 293(28.4) |  |
| IV, n (%) | 131(6.3) | 46(4.5) | 85(8.2) |  |
| LVEF, Mean (SD), % | 41.8(5.6) | 41.7(5.8) | 41.9(5.4) | 0.420 |
| NYHA class |  |  |  | 0.803 |
| I, n (%) | 298(14.4) | 151(14.6) | 147(14.2) |  |
| II, n (%) | 740(35.9) | 367(35.6) | 373(36.1) |  |
| III, n (%) | 919(44.5) | 465(45.1) | 454(44.0) |  |
| IV, n (%) | 107(5.2) | 49(4.7) | 58(5.6) |  |
| Prior cardiovascular surgery, n (%) | 25(1.2) | 11(1.1) | 14(1.4) | 0.546 |
| Elective surgery, n (%) | 1986(96.2) | 993(96.2) | 993(96.2) | 1.000 |
| Preoperative IABP, n (%) | 68(3.3) | 28(2.7) | 40(3.9) | 0.139 |
| Number of graft, Median (quartile) | 4(3, 4) | 4(3, 4) | 4(3,5) | 0.079 |
| Off-pump surgery, n (%) | 1743(84.4) | 874(84.7) | 869(84.2) | 0.761 |
| EuroSCORE |  |  |  | 0.003 |
| 0-2, n (%) | 614 (29.7) | 328 (31.8) | 286 (27.7) |  |
| 3-5, n (%) | 1012 (49.0) | 515 (49.9) | 497 (48.2) |  |
| 6 plus, n (%) | 438 (21.2) | 189 (18.3) | 249 (24.1) |  |

BMI indicate body mass index, CAD, coronary vascular disease; CCS, Canadian Cardiovascular Society; COPD, chronic obstructive pulmonary disease; IABP, intra-aortic balloon pump; LFEF, left ventricular ejection fraction; MI, myocardial infarction; NA, not available; NYHA, New York Heart Association; PTCA, percutaneous transluminal coronary angioplasty; SD, standard deviation.

Table S3 Mortality Ors for Subgroups in Propensity-Matched Group (Non-smokers vs Ex-smokers)

| Subgroup | No. (%) of Events by Group | |  |  |
| --- | --- | --- | --- | --- |
|  | Non-smokers  (n=1731) | Ex-smokers  (n=1731) | OR (95% CI) | *P* Value for Interaction |
| Sex Gender |  |  |  |  |
| Male | 79(4.8) | 67(4.2) | 0.852(0.611-1.189) | 0.120 |
| Female | 11(11.3) | 7(6.0) | 0.498(0.185-1.337) |  |
| Age |  |  |  |  |
| <65y | 40(3.8) | 38(3.4) | 0.883(0.562-1.388) | 0.514 |
| > 65y | 50(7.4) | 36(6.0) | 0.797(0.521-1.242) |  |
| LVEF, % |  |  |  |  |
| > 30 | 82(4.9) | 69(4.1) | 0.829(0.597-1.150) | 0.686 |
| < 30 | 8(17.0) | 5(14.3) | 0.813(0.241-2.737) |  |
| Diabetes Mellitus |  |  |  |  |
| Yes | 31(5.6) | 30(5.4) | 0.959(0.572-1.606) | 0.500 |
| No | 59(5.0) | 44(3.8) | 0.738(0.495-1.101) |  |
| Hypertension |  |  |  |  |
| Yes | 47(5.1) | 44(4.8) | 0.942(0.618-1.435) | 0.342 |
| No | 43(5.4) | 30(3.7) | 0.679(0.421-1.094) |  |
| COPD |  |  |  |  |
| Yes | 3(9.7) | 3(10.3) | 1.077(0.199-5.819) | 0.771 |
| No | 87(5.1) | 71(4.2) | 0.804(0.586-1.112) |  |

CI indicates confidence interval; COPD, chronic obstructive pulmonary disease; LVEF, left ventricular ejection fraction OR, odds ratio.

Table S4 Mortality Ors for Subgroups in Propensity-Matched Group (Non-smokers vs Current smokers)

| Subgroup | No. (%) of Events by Group | |  |  |
| --- | --- | --- | --- | --- |
|  | Non-smokers  (n=1032) | Current Smokers  (n=1032) | OR (95% CI) | *P* Value for Interaction |
| Sex Gender |  |  |  |  |
| Male | 46(4.6) | 29(3.0) | 0.633(0.394-1.016) | 0.007 |
| Female | 5(12.5) | 0(0.0) | 0(0) |  |
| Age |  |  |  |  |
| <65y | 32(4.6) | 17(2.4) | 0.503(0.277-0.915) | 0.839 |
| > 65y | 19(5.7) | 12(3.9) | 0.663(0.316-1.390) |  |
| LVEF, % |  |  |  |  |
| > 30 | 47(4.7) | 28(2.8) | 0.577(0.358-0.929) | 0.323 |
| < 30 | 4(11.8) | 1(4.5) | 0.357(0.037-3.426) |  |
| Diabetes Mellitus |  |  |  |  |
| Yes | 17(4.8) | 10(2.6) | 0.522(0.236-1.155) | 0.922 |
| No | 34(5.0) | 19(3.0) | 0.578(0.326-1.024) |  |
| Hypertension |  |  |  |  |
| Yes | 23(4.2) | 15(2.6) | 0.616(0.318-1.193) | 0.502 |
| No | 28(5.7) | 14(3.0) | 0.510(0.265-0.981) |  |
| COPD |  |  |  |  |
| Yes | 1(5.3) | 0(0.0) | 0(0) | 0.630 |
| No | 50(4.9) | 29(2.9) | 0.566(0.355-0.902) |  |

CI indicates confidence interval; COPD, chronic obstructive pulmonary disease; LVEF, left ventricular ejection fraction OR, odds ratio.
